# Very late-onset Krabbe disease with concomitant dementia: case description and a critical review of the literature

**DOI:** 10.1007/s10072-026-08836-5

**Published:** 2026-02-13

**Authors:** Salvatore Rossi, Alessandra Tessa, Maria Gabriella Vita, Rosellina Russo, Davide Parisi, Fiorella Piemonte, Gianmarco Dalla Zanna, Filippo Maria Santorelli, Gabriella Silvestri

**Affiliations:** 1 Department of Neurosciences, Università Cattolica del Sacro Cuore, Rome, Italy; 2 Molecular Medicine, IRCCS Stella Maris Foundation, Pisa, Italy; 3 UOC Di Neurologia - Dipartimento Di Neuroscienze, Organi Di Senso E Torace, Fondazione Policlinico Universitario Agostino Gemelli IRCCS, Rome, Italy; 4 Advanced Radiology Center (ARC), Department of Radiology and Oncological Radiotherapy, Fondazione Policlinico Universitario Agostino Gemelli IRCCS, 00168 Rome, Italy; 5 Unit of Muscular and Neurodegenerative Diseases, Bambino Gesù Children’s Hospital, IRCCS, Rome, Italy

**Keywords:** Krabbe disease, Adult-onset Krabbe disease, Globoid cell leukodystrophy, GALC, Galactocerebrosidase deficiency

## Abstract

**Background:**

Krabbe disease (KD) is a rare autosomal recessive lysosomal storage disorder caused by pathogenic variants in *GALC*. Despite accounting only for 5% of forms, reports of adult-onset KD cases are increasingly described.

**Methods:**

A female patient manifesting KD after the age of 60 years, presenting with spastic paraplegia and cognitive decline, is described. The scientific literature of KD with onset > 10 years has been extensively reviewed to refine the spectrum of later-onset KD manifestations.

**Results:**

Including ours, we identified 84 KD adolescent/adult-onset patients (mean age at onset 28.7 ± 14.2 years). Most patients had limb spasticity as main characterizing neurological feature (58/84, 70.2%), followed by polyneuropathy (11/ 84, 13.1%), both upper and lower motor neuron signs (2/84, 2.4%), and epilepsy (2/84, 2.4%). Five out of 84 patients (6.0%) were asymptomatic. Most patients had cortico-spinal tracts involvement at brain MRI. The most common pathogenic GALC variants were the c.1901 T > C (18 patients), the c.857G > A (13 patients), and the c.1161 + 6532_polyA + 9kbdel (13 patients).

**Conclusions:**

Complicated spastic paraplegia is the most common manifestation in later-onset KD, rarely with normal brain MRI. KD should be always considered also in cases with very late-onset spastic paraplegia.

**Supplementary Information:**

The online version contains supplementary material available at 10.1007/s10072-026-08836-5.

## Introduction

Krabbe disease (KD, OMIM #245,200), also known as globoid cell leukodystrophy (GLD), is a rare autosomal recessive lysosomal storage disorder (LSD) caused by pathogenic variants in *GALC* (OMIM #606,890), with an estimated prevalence of about 1:100,000 live births [1]. *GALC* codes for beta-galactocerebrosidase (or galactosylceramidase), a lysosomal acid hydrolase involved in the degradation of galactosylceramide, that is a component of the myelin sheat. Its deficiency leads to accumulation within the lysosomes of galactosylceramide and psychosine (also known as galactosylsphingosine), eventually causing damage to myelin in both the central and peripheral nervous systems [2].

Based on the onset of symptoms, KD have been classified into 4 main types, of which the infantile form is the commonest, and it is subdivided in early or late-infantile forms depending on the onset before and after 6 months of age, respectively. Additional clinical subtypes are the juvenile form, manifesting between 2 and 10 years, and the adolescent and adult forms, with onset after 10 and 20 years of age, respectively [3]. Recently, Komatsuzaki et al. proposed a novel classification for KD: early-infantile form (patients with onset of symptoms between 0 and 6 months of age), late-infantile (onset between 7 and 36 months), juvenile/adolescent (onset between 3 and 15 years), and adult-onset (onset after 15 years) [4].

KD occurs in the vast majority of cases in infancy, with rapid neurologic deterioration and progression to death before two years of age. Although data from global disease registries count only approximately 5% of cases with an adolescent/adult-onset phenotype [5], there is an increasing number of clinical reports highlighting the great variability in presenting symptoms and evolution of later-onset KD, often leading to delays in diagnosis [6]. Despite its rarity, more than 300 *GALC* pathogenic variants have been described (https://www.hgmd.cf.ac.uk/ac/gene.php?gene=GALC), and genotype–phenotype correlations are not always clear [7].

Herein, we describe a patient manifesting KD after the age of 60 years and presenting with spastic paraplegia later complicated by cognitive decline. While illustrating the uniqueness of the present case, we also extensively reviewed the scientific literature of KD with onset > 10 years) to further refine the spectrum of manifestations and contextualize the clinical presentation of our patient.

## Materials and methods

Literature review was assessed as it follows: PubMed was searched using the keywords “adult-onset Krabbe disease” and “adult-onset galactocerebrosidase deficiency.” At the time of the writing (August 1 st, 2025), 138 results were found (years of publication from January 1981 to December 2025). Publications were checked for duplicate reports, and only articles in English were considered. Cases with KD onset < 10 years were excluded. Of those, 52 articles were accessible for download and extensively reviewed in order to collect relevant available clinical, biochemical, and/or genetic data.

In total, we identified in the literature 83 adult-onset KD patients from 45 scientific reports with available sufficient information for analysis. In particular, the following variables were collected: sex at birth, age at disease onset (AAO), age at the examination (AE), disease duration (DD), *GALC* pathogenic variants, GALC activity either on leukocytes or fibroblasts derived from skin biopsies, neurological phenotype, neurophysiological data (i.e., electroneurography) and brain MRI findings, and origin of patients. If the origin of the patient was not explicitly stated in the paper, it was attributed to the country of the first author’s institutional affiliation in the respective case description as in other studies [4]. Phenotypic data included the following neurological signs: spastic gait, hyperreflexia, Babinski sign, *pes cavus*, cerebellar ataxia, visual impairment, dysarthria, intellectual disability, and cognitive impairment.

*GALC* variants have traditionally been described on the basis of their amino acid position in the mature enzyme, considering p.M17 the first residue [8]. Current Human Genome Variation Society (HGVS) nomenclature recommendations require proteins to be numbered from the first methionine of the complete 42-residue signal sequence (NM_000153.4) [9]. Here, we reported both nomenclatures of the *GALC* variant as described in the original article, and also the corresponding HGVS nomenclature (which we use throughout this paper).

For statistical analysis, only descriptive techniques were used. Quantitative variables are described using mean and standard deviation (SD), median, minimum (min.), and maximum (max.), interquartile range (IQR). Qualitative variables are summarized as absolute and percentage frequency. Patients with some missing values were included in the study and maintained as missing (is always specified on which denominator percentages are calculated). Of all the variables collected, GALC activity levels was the only variable not summarized for the statistical analysis due to differences in the biochemical methods used for its assessment in the reviewed papers. Statistical analysis was performed using SPSS (Statistical Package for Social Science, IBM SPSS Statistics, version 29.0. IBM Corp.: Armonk, NY, USA).

## Case description

**Fig. 1:**
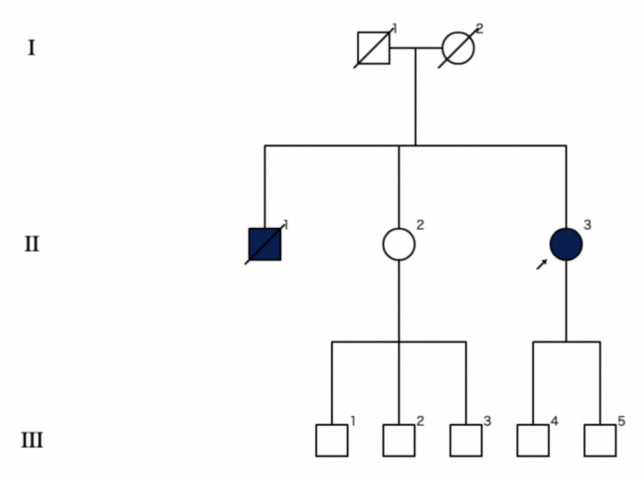
Family tree of the proband (black arrow)

A 66-year-old woman (patient II.3, Fig. 1) came to our observation for gait disturbances with insidious onset and slowly progressive course for the last five years, associated with frequent falls, lower limb cramps, and urinary urgency. Her medical history was remarkable for dyslipidemia in treatment with omega-3 fatty acids and hysterectomy for uterine leiomyomas at 47 years. She was the third child born to non-consanguineous parents both coming from a small town of about 4500 inhabitants in central Italy. She referred that her older brother (subject II.1, Fig. 1), having similar symptoms, was diagnosed with paraplegia of unknown cause and died at 74 years for lung carcinoma. Her 70-year-old sister (subject II.2, Fig. 1) was affected by coxarthrosis, and her neurological evaluation was normal.

At admission, neurological examination of the proband showed spastic paraparesis with no need of walking aids, normal upper limb deep tendon reflexes (DTR) and brisk and symmetric lower limb DTR, bilateral Babinski sign, bilateral *pes cavus,* and no sensory abnormalities. No overt cognitive or cranial nerves abnormalities were found. Spastic paraplegia rating scale (SPRS) total score was 11/52.

Laboratory investigations, including full blood count, kidney and liver function, thyroid function, vitamin B12 and folate levels, HIV and syphilis serology, and an extensive autoantibody profile, were all normal.

**Fig. 2:**
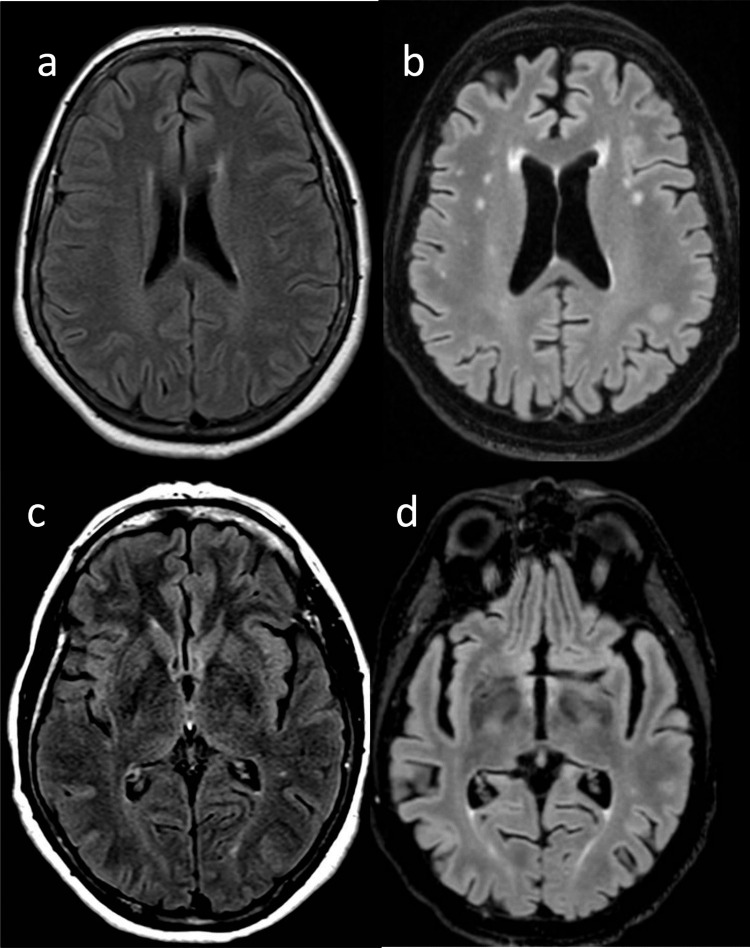
Fluid-attenuated inversion recovery (FLAIR) axial images of brain MRI scans of the proband (a-c: 66 years of age; b-d: 72 years of age). In a and b alterations adjacent to the frontal horns and within the bi-hemispheric white matter are shown, both with progressive increase in time. In d signal alteration of the posterior limb of the internal capsule is shown, which was already present, yet less evident, 8 years before (c)

Brain MRI documented sparse white matter T2-weighted hyperintensities without contrast enhancement (CE) (Fig. 2, panel A-C), and a meningioma stemming from the left greater wing of the sphenoid (maximum diameter 20 mm) with modest mass effect and without indication for a surgical intervention. MRI of the whole spine showed L4-5 anterolisthesis causing lumbar canal stenosis, with normal signal of the spinal cord. Electroneurography was normal, while Motor Evoked and Somato-Sensory Evoked Potentials showed delay of the central motor and somato-sensory conduction related to lower limbs, respectively. Neuropsychological (NPS) assessment by the Mental Deterioration Battery (MDB) [10] showed abnormal results only in one working memory test (backward spatial span) and one visual search test (Multiple Features Targets Cancellation, MFTC), indicative of a mild attention disorder. Corrected Mini-Mental State Examination (MMSE) score was 26.27/30 (Table 1).

**Table 1:** Neuropsychological assessment by the Mental Deterioration Battery of the proband at 66 years (column 2) and 72 years of age (column 3). Corrected scores for age and education are presented in brackets. Pathological scores are highlighted in bold

	*Score (66 years of age)*	*Score (72 years of age)*
Spatial orientation	5/5	**1/5**
Temporal orientation	4/5	**0/5**
Mini-Mental State Examination	25 (26.27)	**15 (17.03)**
*Memory tasks*		
Rey’s auditory verbal learning test (RAVLT) immediate recal	31 (37.1)	**8 (17.55)**
RAVLT delayed recall	9 (10.8)	**0 (0)**
RAVLT forced-choice recognition (correctd -false)	12/15–0/30	**Unable to perform the task**
Rey’s complex figure recall	11.5 (17)	**0 (0)**
Digit span forward	5 (5.39)	5 (5.63)
Digit span backward	3 (3.53)	**0 (0)**
Spatial span forward	4 (4.44)	3 (3.67)
Spatial span backward	**3 (3.06)**	**0 (0)**
*General intelligence task*		
Raven’s colored progressive matrices	20 (23.9)	**9 (14.08)**
Praxis tasks		
Rey’s complex figure copy	27.5 (29.5)	**5.5 (8.28)**
Copy of figures	9 (10.1)	Not assessed
Completion of figures	69 (70)	Not assessed
*Attention and visuo-spatial analysis tasks*		
Multiple features targets cancellation (MFTC) accuracy	**0.808**	**Unable to perform the task**
MFTC false	0 (0)	**Unable to perform the task**
MFTC time	35 (0.94)	**Unable to perform the task**
*Language*		
Phonological verbal fluency	28 (35.9)	25 (34.24)
Categorical verbal fluency (living)	7 (7.29)	3 (3.96)
Categorical verbal fluency (non-living)	7 (7.69)	**4 (5.05)**
Categorical verbal fluency (total)	14 (15.10)	**7 (9.06)**
Naming of pictures of objects	30	**24**
Naming of pictures of verbs	**23**	Not assessed
*Executive functions tasks*		
Stroop test time	29.5 (20.5)	**Unable to perform the task**
Stroop test errors	0 (0)	**Unable to perform the task**

Given the positive family history and the slow progression of the spastic paraparesis, molecular testing for Hereditary Spastic Paraplegias (HSP) was performed. Sanger sequencing and Multiplex ligation-dependent probe amplification (MLPA) for *SPAST* did not show any point mutations, wide rearrangements or exon deletions/duplications. A Next-generation sequencing (NGS) panel targeted for HSPs [11] showed three monoallelic missense variants of uncertain significance: *DYNC1H1*, NM_001376.5, c.11806G > A p.(Val3936Met), *ZFYVE27*, NM_001002261.4, c.185G > A p.(Arg62Gln), *SYNE1*, NM_182961.4, c.4274A > T p.(Glu1425Val). The only variant possibly related to the phenotype was the *ZFYVE27* p.(Arg62Gln), that however was detected also in leukocyte DNA obtained from the healthy sister of the patient.

At 70 years, the patient underwent posterior lumbar arthrodesis for worsening of lumbar canal stenosis, with improvement of lower-limb cramps and urinary urgency, while the spastic gait disturbance kept on worsening.

**Fig. 3:**
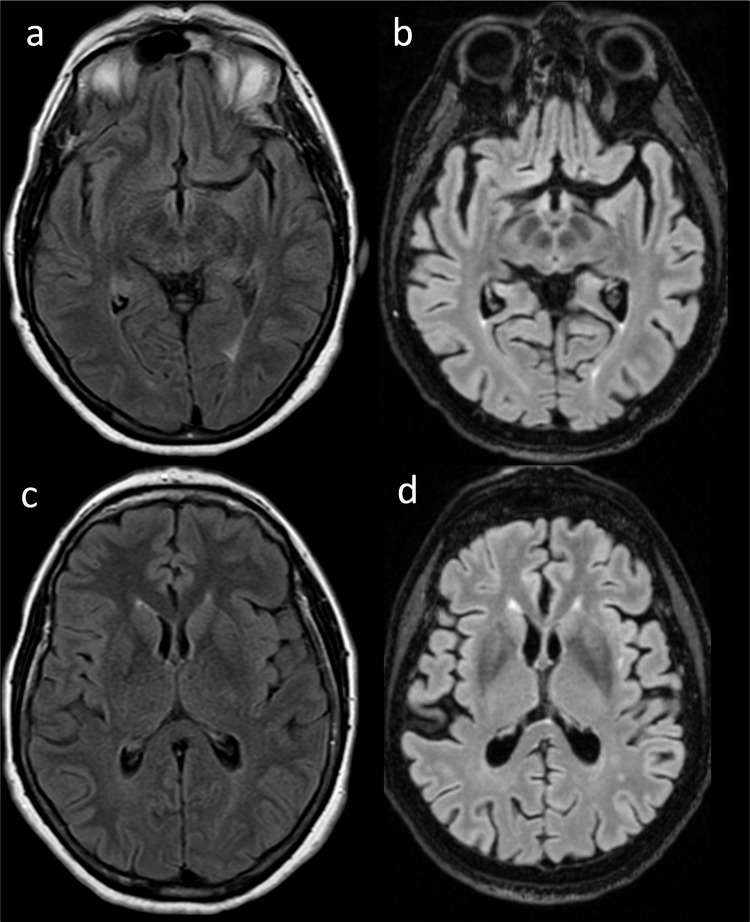
Fluid-attenuated inversion recovery (FLAIR) axial images of brain MRI scans of the proband (a-c: 66 years of age; b-d: 72 years of age), showing progressive parenchymal volume, with enlargement of the Sylvian fissures over time (a-b) and of the supratentorial ventricular system (c-d)

At 72 years, the patient complained of further gait worsening and also of memory problems. Her family relatives reported of occurrence of irritability, apathy, loss of initiative, and progressive loss of autonomy. Her total SPRS score was 18/52. A new brain MRI (Fig. 2, panel B-D) showed worsening of the WM T2-w hyperintensities, mild bilateral T2-w hyperintensity of the posterior internal capsule that re-evaluating the previous MRI scan was already present, signs of global cortical atrophy (Fig. 3), and stability of the meningioma.

A novel NPS assessment documented corrected MMSE = 22.3, global worsening of memory and executive functions, with impairment in tests of episodic verbal memory (i.e., Rey Auditory Verbal Learning Test, RAVLT), visual episodic memory (copy of the Rey-Osterrieth figure), visual attention (MFTC), visual search and cognitive flexibility (MFTC and Trail Making Test, TMT), inhibition of automatic response (Stroop test) (Table 1).

Lumbar puncture (LP) was then performed, showing normal opening pressure, slightly elevated cerebro-spinal fluid (CSF) proteins (47 mg/dl, n.v. 20–40), normal glucose, no cells, and absence of oligoclonal bands. CSF neurodegeneration biomarkers profile documented borderline levels of beta amyloid_1-42_ (600 pg/ml, normal values (n.v.) > 599) and beta amyloid_1–40_ (9956 pg/ml), with reduced ratio amyloid beta 40/42 (0.060, n.v. > 0.069), along with raised total tau (649 pg/ml, n.v. < 404) and phospho-tau (97.7 pg/ml, n.v. < 56.5). Even if an amyloid-PET was not performed, a diagnosis of dementia suggesting Alzheimer’s disease (AD) was made by currently adopted criteria [12], and therapy with memantine 20 mg orally per day and vortioxetine 20 mg per day were initiated. At 74 years, clinical follow-up confirmed progression of cognitive impairment, corrected MMSE score was 17.03.

Suspecting a common genetic etiology for HSP and dementia in our patient, a NGS panel targeted for dementia genes was also performed. This test showed the homozygous *GALC* (NM_000153.3) c.857G > A p.(Gly286Asp) missense variant (previously known as G270D) [13], being classified as pathogenic and already reported in the literature [13–22]. Accordingly, the galactosylceramidase activity, measured on leukocytes by a fluorometric assay, was markedly reduced (3 nmol/mg), if compared to normal range (10–36 nmol/mg) [23]. Finally, a diagnosis of adult-onset KD was made in our proband.

## Literature review

Including our case, we identified 84 KD patients with onset of symptoms > 10 years of age (Supplementary Table 1). Thirty-five were males (41.6%), 45 females (53.5%), and in 4 patients (4.8%) sex was not specified [13].

Out of 84 KD patients, 38 were of Asian descent (45.2%), 31 of European descent (36.9%), 11 were from America (13.1%), 2 from Africa (2.4%), and 2 (2.4%) of mixed ancestry (North Europe and Myanmar). The most represented countries were China (17 patients) and Japan (11 patients), followed by Italy and Canada (6 patients each), Korea (5 patients), United Kingdom (4 patients), India, France, United States of America, Germany (3 patients each). Other countries (Portugal, Iran, Brazil, Sweden, Reunion Islands, Turkey, Morocco, Caribbean Islands, Poland and Belgium) contributed each with one patient.

Mean AAO (n = 79) was 28.7 ± 14.2 years (median 23.0, min. 11.0, max. 66.0, IQR 24.0 years), mean AE was 38.7 ± 15.4 years (median 37.5, min. 12.0, max. 72.0, IQR 26.0 years).

Most patients had spasticity as main characterizing neurological feature (58/84, 70.2%), being spastic paraplegia the most common presentation (50/84, 59.5%), followed by spastic hemiplegia (5/84, 6.0%), and spastic tetraplegia (3/84, 3.6%, Table 2).

**Table 2:** Table summarizing phenotype of the 84 KD patients reviewed in this paper

Main neurological presenting feature	Associated neurological features
Spasticity (58/84, 70.2%) • Spastic paraplegia (50/84, 59.5%) • Spastic hemiplegia (5/84, 6.0%) • Spastic tetraplegia (3/84, 3.6%)	- polyneuropathy 22/43 (51.2%) - cerebellar signs 14/50 (28.0%) - *pes cavus* 11/51 (21.6%) - cognitive impairment 11/54 (20.4%) - dysarthria 10/51 (19.6%) - visual impairment 4/50 (8.0%) - intellectual disability 3/51 (5.9%)
Polyneuropathy (11/84, 13.1%)	- cerebellar signs 1/10 (10.0%) - dysarthria 1/10 (10.0%) - pyramidal signs 2/10 (20.0%) - intellectual disability 1/10 (10.0%) - cognitive impairment 1/10 (10.0%)
Amyotrophic Lateral Sclerosis (3/84, 3.5%)	
Epilepsy (2/84, 2.4%)	
Asymptomatic (5/84, 6.0%)	

Only 14/58 KD patients with spastic paraplegia (24.1%) manifested with a pure form. Other neurological features associated with spasticity among these 58 patients included polyneuropathy in 22/43 (51.2%), cerebellar signs in 14/50 (28.0%), *pes cavus* in 11/51 (21.6%), dysarthria in 10/51 (19.6%), visual impairment in 4/50 (8.0%), and intellectual disability in 3/51 (5.9%). Cognitive impairment was reported in 11/54 (20.4%). No differences were found in terms of age of onset between pure (33.5 ± 12.9 years, n = 14) and complicated forms (27.2 ± 14.6 years n = 44) (p = 0.195 by Mann-Whytney U test).

In out of 50/58 patients brain MRI data were available, the vast majority (47/50, 94.0%) had white matter alterations, mainly involving the corticospinal tracts and/or the corpus callosum and/or the optic radiations. Only 3 (6.0%) had a normal brain MRI.

In 11 patients out of 84, polyneuropathy was instead the main clinical presentation (13.1%). In 5 cases, it was a sensory-motor demyelinating polyneuropathy. Of these 11 KD patients with neuropathy, 7 (63.6%) had a pure neuropathic presentation, while cerebellar signs were reported in 1/10 (10.0%), dysarthria in 1/10 (10.0%), pyramidal signs in 2/10 (20.0%), intellectual disability in 1/10 (10.0%), cognitive impairment in 1/10 (10.0%). In all 7 out 11 patients with brain MRI data available, hyperintensity of posterior white matter was reported.

Five out of 84 patients (6.0%) included in this review, diagnosed with KD after family screening of relatives, were actually asymptomatic (16, 29, 32, and 35 years of age, respectively; for one patient age was not specified [24]). However, 4 of them with available brain MRI data had abnormalities of white matter, especially of the cortico-spinal tracts.

Lastly, 3/84 (3.5%) patients had both upper and lower motor neuron signs, while in 2/84 (2.4%) epilepsy was the neurological onset presentation.

Out of 132 alleles from 68 patients, the most common variants found included the c.1901 T > C (previously known as L618S, n = 23, 17.4%, detected in 18 patients), the c.857G > A (n = 17, 12.9%, from 13 patients), and the c.1161 + 6532_polyA + 9kbdel (n = 13, 9.8%, from 13 patients). All variants found are summarized in Supplementary Table 2.

## Discussion

KD is a rare lysosomal storage disorder that typically presents very early in life as rapid progressing leukodystrophy [3]. As for other rare inherited neurological diseases, with the advent of next-generation sequencing techniques, reports of later-onset forms became more frequent also in KD, allowing to correctly point out to this condition in cases initially misdiagnosed as Amyotrophic Lateral Sclerosis (ALS) [25], multiple sclerosis [14, 26], hereditary spastic paraplegia [27], or Charcot Marie Tooth disease [3, 26, 28].

Here, we reported a very late presentation in a woman diagnosed as a pure hereditary spastic paraplegia with onset after 60 years of age. Albeit rare, such very late age at onset represents an extreme manifestation included in the phenotypic spectrum of KD. Indeed, our literature review revealed two other cases with onset > 60 years. Of note, one was a 72-year-old Moroccan woman similarly showing spastic paraplegia and cognitive decline [18], whereas the other a 60-year-old Japanese woman manifesting with spastic paraplegia and sensory-motor demyelinating polyneuropathy [29].

Our case had not polyneuropathy, although such feature results quite common also in adult-onset individuals (> 50% of cases) in our review. More importantly, her brain MRI showed mild, not-specific signs. Only at age 72 years, when her motor and also cognitive performances had worsened, a follow-up brain MRI scan documented a mild involvement of the cortico-spinal tracts on T2 images. This radiological sign has been proposed a hallmark of adult-onset KD in a recent work conducted on 21 adult-onset KD patients [30], although it is also observed in other neurodegenerative conditions characterized by progressive pyramidal pathway degeneration as ALS, Primary Lateral Sclerosis [31] and adult polyglucosan body disease [32], or other diseases with CNS involvement such as autoimmune central nervous system (CNS) disorders [33], primary CNS lymphoma [34], and Chediak-Higashi syndrome [35].

It is worth noting that brain MRI in patients with KD generally shows alterations of cerebral white matter as highlighted by our literature review, documenting only 3 KD cases with normal neuroimaging. In particular, among these 3 patients, only one case, a 46‐year‐old male with onset two years before of spastic paraplegia, underwent 3 T brain MRI, that was completely normal [36]. The other two patients, two siblings with spastic paraplegia, were studied before 2002, realistically with low-field brain MRI: in one patient the brain MRI was reported as essentially normal, even if a subtle increase in T2 signal in posterior white matter was noted at a follow-up brain MRI, while the other had normal white matter and a non-specific lesion in the right anterior thalamus [27]. Therefore, normal brain MRI in KD patients is possible but very unlikely.

Notably, in our very late-onset KD case, we documented the co-occurrence of cognitive decline resembling AD. In this regard, in our literature review we identified, including our proband, only 13/84 cases (15.5%) [13, 17, 19, 37–39] with variable signs of cognitive impairment as part of the phenotype. Most of the reviewed cases with cognitive involvement did not report any formal NPS assessment [13, 17–19, 38, 39]. Only for one adult-onset KD case, regarding a 22-year-old woman with several episodes of generalized seizure followed by psychotic symptoms, there is a detailed report of her cognitive evaluation, assessed by both MMSE (= 23/30) and the Cambridge Cognitive Examination Chinese version (66/108), along with brain MRI data showing mild brain atrophy [37]. To our knowledge, our case is the first adult-onset KD patient who received deep CSF investigations and detailed NPS assessment.

In fact, out of 13 cases of KD with cognitive impairment, 4 had poor clinical details [13], 3 had KD onset in their second decade and were examined in their third decade [37, 38, 40], 1 had KD onset at 14 years and was examined at 70 years [18], while the other 5 had KD onset in their fifth-to-seventh decade and were examined afterwards [17–19, 39]. Unfortunately, for 9 out of these 13 KD patients, there is mention only of a gradual, not-otherwise-specified cognitive impairment [13, 17, 18, 38]. The further 3 cases, for whom overt dementia was reported, were all over the age of 50 years (a 53-year-old, bedridden male [39], a 70-year-old female [18], and a 63-year-old male patient, respectively [19]), and only for two of them there was indication of development of dementia about 8–10 years after the onset of motor disturbances in their fifties [19, 39].

Given the age at onset of cognitive decline in our patient, figuring out if dementia would be specifically related to KD or a coincidental AD is definitely a challenging matter, in the absence of definitive neuropathology. Supporting a role for beta-galactocerebrosidase deficiency in causing cognitive impairment there is the evidence of cognitive manifestations in the above-mentioned patient with adult-onset KD [37], where a possible co-occurrence of another cause for dementia is unlikely given the young age (22 years). Mechanisms by which neurodegeneration is initiated and promoted in KD include not only CNS accumulation of galactosylceramide and psychosine, but also aberrant folding and subsequent aggregation of mis-folded proteins, which is a finding shared by most neurodegenerative disorders [41]. As a matter of fact, accumulation of the misfolded alpha-synuclein has been demonstrated both in KD animal models and human brains. In the Twitcher mouse, which is the murine model of KD [42], aggregates of alpha-synuclein are almost exclusively neuronal, originating in the medulla and pontine regions, then spreading into the midbrain structures, and eventually affecting the cerebral cortex [43]. Also in brains of human KD patients, alpha-synuclein aggregations have been identified, particularly in the cortex [43], and this might explain the high rate of cognitive impairment in later-onset forms of KD, as many late-onset neurodegenerative disorders accumulate multiple aggregate species, with evidence suggesting that alpha-synuclein and amyloid-beta could mutually promote each other’s accumulation [42, 44].

Moreover, a recent paper by Feo et al. suggested that *GALC* variants even in the heterozygous state may contribute to neurodegeneration, as they were enriched in a cohort of patients affected by various neurodegenerative conditions (mainly atypical parkinsonism) compared to the general population [45]. This is not surprising, as we know that while biallelic pathogenic variants in *GBA1* cause the lysosomal storage disorder Gaucher’s disease, monoallelic variants are a risk factor for Parkinson’s disease development [46]. Therefore, similarly to what happens in Parkinson’s disease patients carrying single *GBA1* variants, it is possible that *GALC* monoallelic pathogenic variants, interacting either with other genetic factors or environmental agents, may induce or maintain neurodegeneration.

From a molecular point of view, the pathogenic *GALC* variant found in our proband, the missense c.857G > A p.(Gly286Asp) previously known as c.809G > A p.G270D, has been described in a similar homozygous state only in one Japanese case with KD onset in the teens, with the authors reporting only that the patient had manifested with spastic paraplegia and mild mental deterioration [13]. The authors also carried out expression studies of the mutant *GALC* cDNA in COS-1 cells, which showed reduced, but not abolished, GALC activity in transfected cells, thus explaining the adult-onset phenotype of the patient [13]. Accordingly, another recent study showed that human oligodendrocytic cell lines carrying the p.(Gly286Asp) variant showed reduced GALC activity between 2 and 7% compared to wild type cells. This intermediate value allowed to classify this variant as “mild”, compared to those variants from infantile-onset KD patients producing less than 2% of wild type GALC activity [47].

Besides these two homozygous cases, fourteen other adult-onset KD cases reported in the literature harbored the c.857G > A p.(Gly286Asp) in compound heterozygosity with other pathogenic variants: the c.1468 T > A p.(Tyr490Asn) [25], the c.349A > G p.(Met117Val) [14], the c.1161 + 6532_polyA + 9kbdel (known also as 30-kb deletion common in Europe) [15, 18, 19, 48], the deletion of exon 17 [16], the c.1075_1084del p.(Lys359Alafs*3) [17], the c.1586C > T p.(Thr529Met) [18], the c.953C > G p.(Pro318Arg) [20], the c.683_694delinsCTC p.(Asn228_Ser232delinsThrPro) [21], and the c.908 + 1G > A [22], respectively. Further supporting a mild pathogenic effect of the c.857G > A p.(Gly286Asp) is the fact that patients harboring such variant along with *GALC* deletions [16, 18, 19, 21, 49] or frameshift variants [17]*,* that virtually may abolish GALC activity, had an adult-onset presentation with relative benign course.

Currently, in some countries a newborn screening for KD is available, and mutational analysis and residual GALC enzyme activity have only limited ability to predict age of disease onset [50]. Having large cohorts of KD patients characterized both in phenotype and genotype is therefore important, as it may help in categorizing a given variant as “mild” or “severe” [7]. This issue is fundamental, as to date the only disease-modifying treatment for KD is represented by hematopoietic stem cell transplantation (HSCT) [51]. Although not a cure, HSCT can prolong life and preserve cognitive skills when performed in presymptomatic infants, but it still exposes transplanted children to considerable morbidity and mortality [7], thus description of other cases with the same genotype may help in defining the detrimental role of variants and guide therapeutic decisions [7].

Besides the c.857G > A that we already discussed, the most frequent pathogenic variant found in our literature cohort was the c.1901 T > G (previously known as L618S), described only in cases of Asian descent, raising the possibility of a founder effect in these populations [24]. It has never been reported in early-infantile cases, but quite frequent in later-onset cases [24]. Supporting a “mild” detrimental effect also for this variant on GALC activity, Shin et al. showed higher enzymatic GALC activity in HEK-293 T cells transfected with the c.1901 T > G compared to cells transfected with variants associated with infantile cases [52].

The other prevalent pathogenic variant in our reviewed cohort was the c.1161 + 6532_polyA + 9kbdel (also known as the 30 kb del), frequent in patients from Europe [39]. This deletion starts in intron 10 and extends to exon 17, eliminating the coding region for the 30‐kDa subunit and 15% of the coding region for the 50‐kDa subunit, giving as a consequence a “severe” effect on GALC activity, as eliminates normal *GALC* transcripts and GALC protein, leading to its complete functional deficiency [39, 48]. Such pathogenic variant causes infantile-onset phenotypes in homozygous carriers, whereas the 13 adult-onset patients of our literature cohort were all compound heterozygous for the 30 kb del and other missense variants.

In conclusion, our report of an additional adult-onset KD patient presenting with spastic paraplegia provided insights on the phenotypic spectrum of the later-onset KD cases. Compared to the most severe infantile KD subtypes [53, 54], the spectrum of phenotypes is wider, making the clinical diagnosis cumbersome. The infantile form starts early with loss of developmental milestones or motor delay, feeding difficulties, irritability, abnormal muscle tone, and progresses rapidly with visual difficulties, apneic episodes, seizures, with a median survival time of 2 years [53]. In the context of such a severe condition, nerve conduction studies are abnormal in almost 90% of patients, and epilepsy occurs in about one-third of patients [54]. On the contrary, later-onset phenotypes may manifest throughout all life, from the second to the seventh decade, and main manifestation is represented by spastic paraplegia. In particular, our case report with critical literature review emphasizes how deep phenotyping might be helpful to address KD diagnosis, as less than 25% of later-onset forms manifest with a pure phenotype, and also in these cases brain MRI scans are rarely normal. In our case, diagnostic delay was caused by the very late onset pure phenotype, and the absence of *GALC* in the NGS panel designed for HSPs, although it included more than 100 HSP-associated genes. Thus, we suggest adding *GALC* in the list of HSP-related genes included in targeted resequencing NGS panels.

Our study has some limitations. First of all, we do not have any brain neuropathology data of the proband herein described. Secondly, data collection from the literature is somehow limited by the inclusion of reports, some published decades ago, for which genotype information were not fully available, or imaging and neurophysiology data were incomplete. Both the rarity and the clinical heterogeneity of presentation of later-onset KD points to the need of a homogeneous, standardized data collection promoted by international research networks, also including objective and patient reported measures, in order to track natural history, phenotype and its correlation with genotype, in view of future disease-modifying therapies other than HSCT suitable for such milder forms.

## Supplementary Information

Below is the link to the electronic supplementary material.

Supplementary file1 (PDF 216 KB)

Supplementary file2 (PDF 57.7 KB)

## Data Availability

The dataset analysed during the current study comes from literature review and is available from the corresponding author on reasonable request.
