# Supplementary material for: Very late-onset Krabbe disease with concomitant dementia: case description and a critical review of the literature
Source: Neurol Sci. 2026 Feb 13;47(3):257. doi: 10.1007/s10072-026-08836-5 (PMC12904878; doi:10.1007/s10072-026-08836-5)
Supplement: Supplementary file 1 — Supplementary file1 (PDF 216 KB) [file 10072_2026_8836_MOESM1_ESM.pdf]

Supplementary Table 1. Table showing clinical, molecular and imaging findings collected from Krabbe disease patients by the literature review. Abbreviations: CC: corpus callosum; CST: cortico-spinal tracts; n.v.: normal values, UK: United Kingdom; WM: white matter.

Origin	Main presentation	Sex	Age at onset	Age at examination	Variant 1 (as reported in the original paper)	Variant 1 (NM_000153.4)	Variant 2 (as reported in the original paper)	Variant 2 (NM_000153.4)	GALC activity	Brain MRI findings	Reference
Germany	Spastic paraplegia	M	44	46	ex 11-17 (30 kb) del	c.1161+6532_polyA+9kbdel / p.(?)	c.334A>G / p.(Thr112Ala)	c.334A>G / p.(Thr112Ala)	0.29% (n.v. >2%)	Normal	Machtel et al.[1]
China	Spastic paraplegia	M	46	49	c.599C>A / p.S200X	c.599C>A / p.(Ser200Ter)	c.1901T>C / p.L634S	c.1901T>C / p.(Leu634Ser)	1 nmol/17h/mg Pr (n.v. 19-68.2)	T2 hyperintensity of the CST	Zhang et al. 2021 [2]
China	Spastic paraplegia	M	20		c.1901T>C / p.L634S	c.1901T>C / p.(Leu634Ser)	c.1901delT / p.L634X	c.1901delT / p.(Leu634Ter)		T2 hyperintensity of the CST	Zhang et al.
China	Spastic paraplegia	M	43		c.1901T>C / p.L634S	c.1901T>C / p.(Leu634Ser)				T2 hyperintensity of the CST	Zhang et al.
UK	Spastic paraplegia	M	11	33	IVS10DEL30	c.1161+6532_polyA+9kbdel / p.(?)			undetectable	Normal	Bajai et al.[3]
UK	Spastic paraplegia	M	31	32					0.04 nmol/h/mg (n.v. 0.51-3.8)	Normal	Bajai et al.
Canada	Cerebellar ataxia	M	18	56	c.334A>G / p.Thr112Ala	c.334A>G / p.(Thr112Ala)	c.592G>A / p.Glu198Lys	c.592G>A / p.(Glu198Lys)		global atrophy of posterior fossa structures	Shao et al. [4]
Canada	Cerebellar ataxia	M	20	60	c.334A>G / p.Thr112Ala	c.334A>G / p.(Thr112Ala)	c.592G>A / p.Glu198Lys	c.592G>A / p.(Glu198Lys)		T2 hyperintensity of the CST, hyperintensity of the parieto-occipital regions and the body of the CC, mild cerebellar and brainstem atrophy	Shao et al.
Canada	Cerebellar ataxia	F	15	58	c.334A>G / p.Thr112Ala	c.334A>G / p.(Thr112Ala)	c.592G>A / p.Glu198Lys	c.592G>A / p.(Glu198Lys)		cerebellar and parietal lobes atrophy	Shao et al.
Canada	Cerebellar ataxia	M	15		c.334A>G / p.Thr112Ala	c.334A>G / p.(Thr112Ala)	c.592G>A / p.Glu198Lys	c.592G>A / p.(Glu198Lys)		Not reported	Shao et al.
Japan	Polyneuropathy	F	14	16	G284D	c.851G>A / p.(Gly284Asp)	I82M+I305V	c.246A>G / p.(Ile82Met) + c.913A>G / p.(Ile305Val)	0.03 nmol/h/mg (n.v. not reported)	T2 hyperintensities of the deep posterior WM	Fukazawa et al. [5]
China	Spastic paraplegia	M	20	20	c.1901delT / p.L634X	c.1901delT / p.(Leu634Ter)	c.1901T>C / p.L634S	c.1901T>C / p.(Leu634Ser)	1.3 nmol/g/min (n.v. >17.2)	T2 hyperintensity of the CST	Zhang et al.2018 [6]
China	Asymptomatic	M			c.1901T>C / p.L634S	c.1901delT / p.(Leu634Ter)	c.1901T>C / p.L634S	c.1901T>C / p.(Leu634Ser)	3.5 nmol/g/min (n.v. >17.2)	T2 hyperintensity of the CST and optic radiations	Zhang et al.2018
China	Spastic paraplegia	F	37	40	c.1658G>A / p.G553E	c.1658G>A / p.(Gly553Glu)	c.1901T>C / p.L634S	c.1901T>C / p.(Leu634Ser)	0.7 nmol/17h/mg protein (n.v. >12)	T2 hyperintensities of the posterior limb of internal capsule and optic radiations	He et al. [7]
Japan	Spastic paraplegia		15		I66M + I289V polymorphism	c.246A>G / p.(Ile82Met) + c.913A>G / p.(Ile305Val)	I66M + I289V polymorphism	c.246A>G / p.(Ile82Met) + c.913A>G / p.(Ile305Val)	0.15 nmol/h (n.v. 3-6 nmol/h)	T2 hyperintensity of the CST	Furuya et al. [8]
Japan	Spastic paraplegia		15		I66M + I289V polymorphism	c.246A>G / p.(Ile82Met) + c.913A>G / p.(Ile305Val)	I66M + I289V polymorphism	c.246A>G / p.(Ile82Met) + c.913A>G / p.(Ile305Val)	0.15 nmol/h (n.v. 3-6 nmol/h)	T2 hyperintensity of the CST	Furuya et al.
Japan	Spastic paraplegia		15		G270D	c.857G>A / p.(Gly286Asp)	G270D	c.857G>A / p.(Gly286Asp)	0.29 nmol/h (n.v. 3-6 nmol/h)	T2 hyperintensity of the CST	Furuya et al.
Japan	Spastic paraplegia		15		ex 6 del (535-573del)	exon 6 deletion (c.583_621del) / p.(?)	L618S	c.1901T>C / p.(Leu634Ser)	0.33 nmol/h (n.v. 3-6 nmol/h)	T2 hyperintensity of the CST	Furuya et al.
India	Spastic paraplegia	F	23	29	ex 11-17 (30 kb) del	c.1161+6532_polyA+9kbdel / p.(?)	c.1850C>T / p.T617M	c.1898C>T / p.(Thr633Met)	undetectable	T2 hyperintensity of the CST, hyperintensity of isthmus of the CC, atrophy of the right precentral gyrus	Farina et al.[9]
India	Spastic paraplegia	M	23	27	ex 11-17 (30 kb) del	c.1161+6532_polyA+9kbdel / p.(?)	c.1850C>T / p.T617M	c.956A>G / p.(Tyr319Cys)	0.06 nmol/mg/h (n.v >2.7)	T2 hyperintensity of the CST, hyperintensity of isthmus of the corpus callosum	Farina et al.
Japan	Spastic paraplegia	F	60		p.G496S	c.1534G>A / p.(Gly512Ser)	p.G596S	c.1912 G>A / p.(Gly638Ser)	0.03 nmol/h/mg (n.v. > 2.1)	T2 hyperintensity of the CST	Tokushige et al.[10]
China	Polyneuropathy	F	17	25	c.1901T>C / p.Leu634Ser	c.1901T>C / p.(Leu634Ser)	c.1901T>C / p.Leu634Ser hom	c.1901T>C / p.(Leu634Ser)	11.63 nmol/17h/mh (n.v. 29.46-34.4)	T2 hyperintensity of the CST	Sun et al. [11]
China	Spastic paraplegia	F	17	27	c.1901T>C / p.Leu634Ser	c.1901T>C / p.(Leu634Ser)	c.1901T>C / p.Leu634Ser hom	c.1901T>C / p.(Leu634Ser)	11.65 nmol/17h/mh (n.v. 29.46-34.4)	T2 hyperintensity of the CST, enlargement of all cerebral ventricles	Sun et al.
Italy	Amyotrophyc Lateral Sclerosis	M	45	47	c.857G>A / p.G286D	c.857G>A / p.(Gly286Asp)	c.1468T>A / p.Y490N	c.1468T>A / p.Tyr490Asn		T2 hyperintensity of the CST, periventricular WM, and semioval centres	Iacono et al. [12]
China	Psychosis and epilepsy	F	13	22	c.1901T>C / p.L634S hom	c.1901T>C / p.(Leu634Ser)	c.1901T>C / p.L634S hom	c.1901T>C / p.(Leu634Ser)	3.3 nmol/17h/mg (n.v. >12.7 nmol/17h/mg)	mild brain atrophy, especially in frontal lobes	Xia et al. [13]
UK	Spastic paraplegia	M	53	67	c.326C>T / p.Thr109Ile	c.326C>T / p.(Thr109Ile)	c.391T>C / p.Trp131Arg	c.391T>C / p.(Trp131Arg)	0.03 nmol/h/mg (n.v. 0.4-4)	T2 hyperintensity of the CST and parietal WM	McCarron et al. [14]

China	Spastic paraplegia	F	29	31	c.1911+1_1911+5delGTAAG	c.1911+1_1911+5del / p.(?)	c.2041G>A / p.V681M	c.2041G>A / p.Val681Met	0.061 nmol/h/mg (n.v. 0.6-3.29)	T2 hyperintensity of the periventricular and subcortical WM, enlargement of the ventricles, grey matter atrophy	Yang et al. [15]
China	Epilepsy	F	22	22	c.1901T>C / p.Leu634Ser hom	c.1901T>C / p.(Leu634Ser)	c.1901T>C / p.Leu634Ser hom	c.1901T>C / p.(Leu634Ser)	3.3 nmol/17h/mg (n.v. >12.7 nmol/17h/mg)	global brain atrophy, right occipital ribbon sign	Wang et al. [16]
China	Spastic paraplegia	F	23	25	c.1901T>C / p.L634S	c.1901T>C / p.(Leu634Ser)	c.749T>C / p.I250T	c.749T>C / p.Ile250Thr	0.1 nmol/17h/mg protein (n.v. 19-68.2 nmol/17h/mg protein)	T2 hyperintensity of the CST and parietal and occipital WM	Zhang et al. 2021[17]
China	Spastic paraplegia	F	21	22	c.1901T>C / p.L634S	c.1901T>C / p.(Leu634Ser)	c.283_284del	c.283_284del / p.(Leu95Alafs*10)	1.1 nmol/17h/mg protein (n.v. 19-68.2 nmol/17h/mg protein)	T2 hyperintensity of the CST and parietal and occipital WM	Zhang et al. 2021
China	Spastic paraplegia	F	29	39	c.1901T>C / p.L634S	c.1901T>C / p.(Leu634Ser)	c.908+5G>A	c.908+5G>A	2.76 nmol/17h/mg (n.v. 29.46-34.40 nmol/17h/mg protein)	T2 hyperintensity of the CST and corona radiata	Su et al. 2023 [18]
Canada	Spastic paraplegia	F	20	35	c.857G>A / p.G286D	c.857G>A / p.(Gly286Asp)	c.349A>G / p.M117V	c.349A>G / p.(Met117Val)	0.2 nmol/h/mg (n.v. 2.1-10.44)	T2 hyperintensity of the CST, cerebellar peduncles, periventricular WM	Sharp et al [19]
Japan	Spastic paraplegia	F	38	51	c.T1853C	c.1901T>C / p.(Leu634Ser)	c.T1853C	c.1901T>C / p.(Leu634Ser)	0.177 nmol/mg/h (n.v. 1.93-5.58)	T2 hyperintensity of the CST, centrum semiovale, atrophy of the splenium of the CC	Satoh et al. [20]
Portugal	Spastic paraplegia	M	27	33	c.884A>C / p.N295T	c.884A>C / p.(Asn295Thr)	c.1655T>C / p.I552T	c.1655T>C / p.(Ile552Thr)	0.14 nmol/h/mg (n.v. 0.71-3.59)	T2 hyperintensity of the CST and CC	Duraes et al [21]
Japan	Polyneuropathy	F	51	55	c.246A>G / p.I82M hom	c.246A>G / p.(Ile82Met)	c.246A>G / p.I82M hom	c.246A>G / p.(Ile82Met)	<5.2 nmol/mg/17h (n.v. 29.8-140)	T2 hyperintensity of the optic radiations and periventricular WM	Adachi et al.[22]
Italy	Spastic paraplegia	M	37	39					reduced	T2 hyperintensity of the CST	Turazzini et al [23]
Italy	Asymptomatic	M		29					reduced	T2 hyperintensity of the CST	Turazzini et al
Japan	Spastic hemiplegia	F	20	24					0.1 nmol/h/mg (n.v. >1)	T2 hyperintensity of the CST and CC	Mamada et al. [24]
China	Spastic paraplegia	M	27	37	c.1899delG / p.T633Tfs*2	c.1899del /p.(Leu634Ter)	c.1586C>T / p.T529M	c.1586C>T / p.(Thr529Met)	1.04 nmol/17h/mg (n.v. >18)	T2 hyperintensity of the CST, optic radiations and CC	Zhong et al [25]
China	Asymptomatic	M		35	c.1899delG / p.T633Tfs*2	c.1899del /p.(Leu634Ter)	c.1586C>T / p.T529M	c.1586C>T / p.(Thr529Met)		Not reported	Zhong et al
China	Spastic tetraplegia	M	30	39	c.461C>A / p.P154H	c.461C>A / p.(Pro154His)	c.1065G>C / p.W355C	c.1065G>C / p.(Trp355Cys)		T2 hyperintensity of the CST, optic radiations and CC	Zhong et al
Brazil	Asymptomatic	F		32	c.1065G>T / p.Trp355Cys	c.1065G>T/ p.(Trp355Cys)	c.977C>T / p.Thr326Met	c.977C>T / p.(Thr326Met)		T2 hyperintensity of the CST, optic radiations and CC, mild atrophy	Paiva et al [26]
UK	Amyotrophyc Lateral Sclerosis	M	43	48	502T deletion (30-kb deletion)	c.1161+6532_polyA+9kbdel / p.(?)	did not find the other mutation		0.008 U/g (n.v. 0.03-0.12)	T2 diffuse hyperintensity of the WM	Henderson et al [27]
Japan	Polyneuropathy	F	20	22					0.17 nmol/(mg*h) (n.v. 1.75-8.23)	T2 hyperintensity of the CST	Hiyama et al [28]
Japan	Polyneuropathy	F	30	50					0.053 nmol/(mg*h) (n.v. 1.75-8.23)	cervical spinal cord atrophy	Hiyama et al
African-American	Spastic hemiplegia	M	16	17	c.G283A / p.Gly95Ser	c.331G>A / p.(Gly95Ser)	c.G147C	c.195G>C / p.(Gly65Gly)	0.09 nmol/mg protein/hr (n.v. 1-8)	T2 hyperintensity of the frontal and parietal lobe WM	Bernardini et al [29]
African-American	Asymptomatic	F		16	c.G283A / p.Gly95Ser	c.331G>A / p.(Gly95Ser)	c.G147C	c.195G>C / p.(Gly65Gly)	0.08 nmol/mg protein/hr (n.v. 1-8)	T2 hyperintensity of the frontal and parietal lobe WM	Bernardini et al
Italy	Polyneuropathy	M	47	59	ex 11-17 (30 kb) del	c.1161+6532_polyA+9kbdel / p.(?)	c.G809A	c.857G>A / p.(Gly286Asp)	very low	T2 diffuse hyperintensity of the WM	Sabatelli et al [30]
Italy	Amyotrophyc Lateral Sclerosis	M	41							Not reported	Sabatelli et al
Italy	Polyneuropathy	M	45							Not reported	Sabatelli et al
Canada	Spastic paraplegia	F	20	42					0.2 nmol/h/mg	T2 hyperintensity of the CST	Laule et al.[31]
Italy	Spastic paraplegia	F	22	33	ex 11-17 (30 kb) del	c.1161+6532_polyA+9kbdel / p.(?)	p.Thr617Met	c.1898C>T / p.(Thr633Met)	0.53 nmol/mg*h (n.v.>2.7)	T2 hyperintensity of the CST and CC	Selleri et al [32]
Italy	Spastic paraplegia	M	12	35	ex 11-17 (30 kb) del	c.1161+6532_polyA+9kbdel / p.(?)	p.Thr617Met	c.1898C>T / p.(Thr633Met)		T2 hyperintensity of the CST	Selleri et al

Sweden	Spastic paraplegia	F	23	28	c.C1543T hom (+ T1637C polim)	c.1591C>T / p.(Arg531Cys) +c.1685T>C / p.(Ile562Thr)	c.C1543T hom (+ T1637C polim)	c.1591C>T / p.(Arg531Cys) +c.1685T>C / p.(Ile562Thr)	0.07 mckat/kg cell protein (0.44-1.46)	T2 hyperintensity of the CST, thin CC with increased signal intensity, atrophy of the spinal cord	Wang et al. [33]
Korea	Polyneuropathy	M	15	20	exon 17 del	exon 17 deletion	c.857G>A	c.857G>A / p.(Gly286Asp)	0.096 nmol/h/mg protein (n.v. >0.3)	Not reported	Hwang et al. [34]
Korea	Spastic paraplegia	F	34	45	c.1901T>C	c.1901T>C / p.(Leu634Ser)	c. 760T>C	c.760T>C / p.(Tyr254His)	5.4 nmol/h/mg/protein (n.v. 24-82)	T2 hyperintensity of the CST, splenium CC, optic radiations	Hwang et al
Korea	Polyneuropathy	F	34	39	c.1901T>C	c.1901T>C / p.(Leu634Ser)	c.952C>G	c.952C>G / p.(Pro318Ala)		T2 hyperintensity of the periventricular WM and dentate nuclei	Hwang et al
USA	Polyneuropathy	M	45	53	c.A286G (Thr96Ala)	c.334A>G / p.(Thr112Ala)	c.A512T (Asp171Val) + other polymorphisms	c.560A>T / p.(Asp187Val)	0.03 (n.v. >4.2 nmol/hr/mg protein)	T2 hyperintensity of the parietal, occipital and superior temporal lobes	Luzi et al. [35]
Italy	Spastic paraplegia	F	37	44	c.121G>A	c.169G>A / p.(Gly57Ser)	c.121G>A	c.169G>A / p.(Gly57Ser)	0.8 nmol/h/mg (n.v. 2-6)	T2 hyperintensity of the WM (more accentuated in the parietal region), slight cortical atrophy	Malandrini et al[36]
Italy	Spastic hemiplegia	M	53	57	c.809G>A / p.G270D + polimorphism 1637T>C (I546T)	c.857G>A / p.(Gly286Asp) + c.1685T>C / p.Ile562Thr			1 nmol/h/mg (n.v. 2-6)	T2 hyperintensity of the periventricular WM, cortico-subcortical atrophy	Malandrini et al
Italy	Spastic paraplegia	F	42	54	c.809G>A / p.G270D	c.857G>A / p.(Gly286Asp)	c.1026del10 Gly362fs	c.1075_1084del / p.(Lys359Alafs*3)	1.1 nmol/h/mg (n.v. 2-6)	T2 hyperintensity of the CST	Malandrini et al
Italy	Cerebellar ataxia	F	23	41	G41S	c.169G>A / p.(Gly57Ser)	G41S	c.169G>A / p.(Gly57Ser)	2%	T2 hyperintensity of the CST	Fiumara et al[37]
Italy	Spastic tetraplegia	F	23	47	G41S	c.169G>A / p.(Gly57Ser)	G41S	c.169G>A / p.(Gly57Ser)	1%	T2 hyperintensity of the WM of CST, parieto-occipital areas, corpus callosum	Fiumara et al
Reunion Islands	Spastic paraplegia	F	12	24	30 kb del	c.1161+6532_polyA+9kbdel / p.(?)	p.Y303C	c.956A>G / p.(Tyr319Cys)	only pooled data available	T2 hyperintensity of the CST, splenium of the CC, optic radiations	Debs et al. [38]
France	Spastic paraplegia	F	14	70	p.G270D	c.857G>A / p.(Gly286Asp)	p.T513M	c.1586C>T / p.(Thr529Met)	only pooled data available	only pooled data available	Debs et al.
France	Spastic tetraplegia	F	22	40	p.T96A	c.334A>G / p.(Thr112Ala)	p.P138R	c.461C>G / p.(Pro154Arg)	only pooled data available	only pooled data available	Debs et al.
Italy	Spastic paraplegia	F	24	25	30 kb del	c.1161+6532_polyA+9kbdel / p.(?)	p.G622S	c.1912 G>A / p.(Gly638Ser)	only pooled data available	only pooled data available	Debs et al.
Turkey	Spastic paraplegia	F	25	56	p.T51I	c.200C>T / p.(Thr67Ile)	p.K123del	c.415_417del / p.(Lys139del)	only pooled data available	only pooled data available	Debs et al.
France	Polyneuropathy	F	50	54	30 kb del	c.1161+6532_polyA+9kbdel / p.(?)	p.G270D	c.857G>A / p.(Gly286Asp)	only pooled data available	only pooled data available	Debs et al.
Caribbean Islands	Spastic paraplegia	M	52	58	ND				only pooled data available	only pooled data available	Debs et al.
Morocco	Spastic paraplegia	F	66	72	p.G49G hom	c.195G>C / p.(Gly65Gly)	p.G49G hom	c.195G>C / p.(Gly65Gly)	only pooled data available	only pooled data available	Debs et al.
Italy	Spastic paraplegia	F	38	42					0.22 nmol/h mg protein (<10%)	T2 hyperintensity of the parieto-occipital WM	Romano et al[39]
Germany	Spastic paraplegia	M	50	62	30 kb del	c.1161+6532_polyA+9kbdel / p.(?)	c.809G>A / p.270gly>asp	c.857G>A / p.(Gly286Asp)	2.19 pmol/h/10^6 cells (11.5%)	global brain atrophy, moderate WM involvement	Harzer et al. [40]
Germany	Spastic paraplegia	M	50	63	30 kb del	c.1161+6532_polyA+9kbdel / p.(?)	c.809G>A / p.270Gly>Asp	c.857G>A / p.(Gly286Asp)	2.66 pmol/h/10^6 cells (14%)		Harzer et al.
North european and Myanmar ancestry	Spastic paraplegia	F	17	19					0.05 (n.v. >1.1 nmol/mg/protein h)	Occipital WM lesions	Kolodny et al. [41]
North european and Myanmar ancestry	Spastic hemiplegia	F	18	18					0.09	Posterior fronto-parietal WM alterations	Kolodny et al.
Poland	Spastic hemiplegia	M	13	20					0.05	Posterior WM abnormalities	Kolodny et al.
Belgium	Spastic paraplegia	F	20						reduced activity	T2 hyperintensity of the CST, splenium corpus callosum, optic radiations	Demaerel et al. [42]
Italy	Spastic paraplegia	M	26	30	p.G286D	c.857G>A / p.(Gly286Asp)	p.P318R	c.953C>G / p.(Pro318Arg)	5% of n.v.	WM alterations	Tappino et al [43]

Korea	Spastic paraplegia	F	30	38	c.857G>A / p.G286D	c.857G>A / p.(Gly286Asp)	c.683_694delinsCTC / p.N228_S232delinsTP	c.683_694delinsCTC / p.(Asn228_Ser232delinsThrPro)	5.1 nmol/hr/mg protein	T2 hyperintensity of the CST	Lim et al. [44]
Korea	Spastic paraplegia	F	12	12	c.1687A>T / p.K563*	c.1687A>T / p.(Lys563Ter)	c.1901T>C / p.L634S	c.1901T>C / p.(Leu634Ser)	1.9 nmol/hr/mg protein	T2 hyperintensity of the CST, optic radiations	Lim et al.
Iran	Spastic paraplegia	F	11	20	c.908+1G>A	c.908+1G>A	c.857G>A / p.Gly286Asp	c.857G>A / p.(Gly286Asp)	4 pmol/spot (n.v. 32-500)	T2 hyperintensity of the CST	Africa et al. [45]
India	Spastic paraplegia	M	13	37					0.06 nmol/mg/h (n.v 0.22-3.8)	Hyperintensities of posterior WM and corpus callosum	Kaopor et al. [46]
Italy	Spastic paraplegia	F	61	72	c.857G>A / p.Gly286Asp	c.857G>A / p.(Gly286Asp)	c.857G>A / p.Gly286Asp	c.857G>A / p.(Gly286Asp)	3 nmol/mg, n.v. 10-36	T2 mild hyperintensitiy of the CST	This report, Rossi et al.

1. Mächtel R, Dobert J-P, Hehr U, et al (2024) Late-onset Krabbe disease presenting as spastic paraplegia - implications of GCase and CTSB/D. Ann Clin Transl Neurol 11:1715–1731. https://doi.org/10.1002/acn3.52078

2. Zhang T, Yan C, Liu Y, et al (2021) Late-Onset Leukodystrophy Mimicking Hereditary Spastic Paraplegia without Diffuse Leukodystrophy on Neuroimaging. Neuropsychiatr Dis Treat 17:1451–1458. https://doi.org/10.2147/NDT.S296424

3. Bajaj NPS, Waldman A, Orrell R, et al (2002) Familial adult onset of Krabbe's disease resembling hereditary spastic paraplegia with normal neuroimaging. J Neurol Neurosurg Psychiatry 72:635–638. https://doi.org/10.1136/jnnp.72.5.635

4. Shao Y-H, Choquet K, La Piana R, et al (2016) Mutations in GALC cause late-onset Krabbe disease with predominant cerebellar ataxia. Neurogenetics 17:137–141. https://doi.org/10.1007/s10048-016-0476-2

5. Fukazawa R, Takeuchi H, Oka N, et al (2021) Adult Krabbe Disease That Was Successfully Treated with Intravenous Immunoglobulin. Intern Med 60:1283–1286. https://doi.org/10.2169/internalmedicine.6094-20

6. Zhang T, Yan C, Ji K, et al (2018) Adult-onset Krabbe disease in two generations of a Chinese family. Ann Transl Med 6:174. https://doi.org/10.21037/atm.2018.04.30

7. He Z, Pang X, Bai J, et al (2022) A novel GALC gene mutation associated with adult-onset Krabbe disease: a case report. Neurocase 28:314–319. https://doi.org/10.1080/13554794.2022.2083518

8. Furuya H, Kukita Y, Nagano S, et al (1997) Adult onset globoid cell leukodystrophy (Krabbe disease): analysis of galactosylceramidase cDNA from four Japanese patients. Hum Genet 100:450–456. https://doi.org/10.1007/s004390050532

9. Farina L, Bizzi A, Finocchiaro G, et al (2000) MR imaging and proton MR spectroscopy in adult Krabbe disease. AJNR Am J Neuroradiol 21:1478–1482

10. Tokushige S, Sonoo T, Maekawa R, et al (2013) Isolated pyramidal tract impairment in the central nervous system of adult-onset Krabbe disease with novel mutations in the *GALC* gene. Brain and Development 35:579–581. https://doi.org/10.1016/j.braindev.2012.08.004

11. Sun Y, Zheng J, He L, et al (2025) Late-Onset Krabbe Disease: Case Report of Two Patients in a Chinese Family and Literature Review. Mol Genet Genomic Med 13:e70065. https://doi.org/10.1002/mgg3.70065

12. Iacono S, Del Giudice E, Leon A, et al (2022) A novel compound heterozygous mutation in GALC associated with adult-onset Krabbe disease: case report and literature review. Neurogenetics 23:157–165. https://doi.org/10.1007/s10048-021-00682-1

13. Xia Z, Wenwen Y, Xianfeng Y, et al (2020) Adult-onset Krabbe disease due to a homozygous GALC mutation without abnormal signals on an MRI in a consanguineous family: A case report. Mol Genet Genomic Med 8:e1407. https://doi.org/10.1002/mgg3.1407

14. McCarron EP, Oldham A, Herwadkar A, et al (2025) Natural History and Diagnostic Findings in an Adult Man Diagnosed With Attenuated Krabbe Disease. Am J Med Genet A 197:e64031. https://doi.org/10.1002/ajmg.a.64031

15. Yang Y, Ren X, Xu Q, et al (2013) Four novel GALC gene mutations in two Chinese patients with Krabbe disease. Gene 519:381–384. https://doi.org/10.1016/j.gene.2013.02.010

16. Wang Y, Wang S-Y, Li K, et al (2022) Adult-onset Krabbe disease presenting with progressive myoclonic epilepsy and asymmetric occipital lesions: A case report. Front Neurol 13:1010150. https://doi.org/10.3389/fneur.2022.1010150

17. Zhang C, Liu Z, Dong H (2021) Two Cases of Female Chinese Adult-Onset Krabbe Disease with One Novel Mutation and a Review of Literature. J Mol Neurosci 71:1185–1192. https://doi.org/10.1007/s12031-020-01742-1

18. Su Y, Wei L, Wang L, et al (2024) Splicing mutations of GALC in adult patient with adult-onset Krabbe disease: case report and review of literature. Neurocase 30:63–67. https://doi.org/10.1080/13554794.2024.2354541

19. Sharp ME, Laule C, Nantel S, et al (2013) Stem cell transplantation for adult-onset krabbe disease: report of a case. JIMD Rep 10:57–59. https://doi.org/10.1007/8904_2012_203

20. Satoh JI, Tokumoto H, Kurohara K, et al (1997) Adult-onset Krabbe disease with homozygous T1853C mutation in the galactocerebrosidase gene. Unusual MRI findings of corticospinal tract demyelination. Neurology 49:1392–1399. https://doi.org/10.1212/wnl.49.5.1392

21. Durães J, Salsano E, Macário M do C (2021) Adult-Onset Krabbe Disease. Neurology Clinical Practice 11:e15–e17. https://doi.org/10.1212/CPJ.0000000000000780

22. Adachi H, Ishihara K, Tachibana H, et al (2016) Adult-onset Krabbe disease presenting with an isolated form of peripheral neuropathy. Muscle Nerve 54:152–157. https://doi.org/10.1002/mus.25067

23. Turazzini M, Beltramello A, Bassi R, et al (1997) Adult onset Krabbe's leukodystrophy: a report of 2 cases. Acta Neurol Scand 96:413–415. https://doi.org/10.1111/j.1600-0404.1997.tb00308.x

24. Mamada N, Nakamagoe K, Shioya A, et al (2016) Adult-onset Krabbe disease presenting as acute hemiparesis and progressive demyelination detected by diffusion-weighted imaging. J Neurol Sci 367:326–328. https://doi.org/10.1016/j.jns.2016.06.026

25. Zhong J, Jiang F, Yang H, et al (2020) Novel GALC Mutations Cause Adult-Onset Krabbe Disease With Myelopathy in Two Chinese Families: Case Reports and Literature Review. Front Neurol 11:830. https://doi.org/10.3389/fneur.2020.00830

26. Paiva ARB, Fonseca Neto RE, Afonso CL, et al (2022) Incidental magnetic resonance imaging findings leading to an unusual diagnosis: Adult onset Krabbe disease. Eur J Neurol 29:1859–1862. https://doi.org/10.1111/ene.15298

27. Henderson RD, MacMillan JC, Bradfield JM (2003) Adult onset Krabbe disease may mimic motor neurone disease. J Clin Neurosci 10:638–639. https://doi.org/10.1016/s0967-5868(02)00302-8

28. Hiyama T, Masumoto T, Hara T, et al (2016) Enlargement of the brachial plexus on magnetic resonance imaging: a novel finding in adult-onset Krabbe disease. BJR Case Rep 2:20150213. https://doi.org/10.1259/bjrcr.20150213

29. Bernardini GL, Herrera DG, Carson D, et al (1997) Adult-onset Krabbe's disease in siblings with novel mutations in the galactocerebrosidase gene. Ann Neurol 41:111–114. https://doi.org/10.1002/ana.410410119

30. Sabatelli M, Quaranta L, Madia F, et al (2002) Peripheral neuropathy with hypomyelinating features in adult-onset Krabbe's disease. Neuromuscul Disord 12:386–391. https://doi.org/10.1016/s0960-8966(01)00285-1

31. Laule C, Vavasour IM, Shahinfard E, et al (2018) Hematopoietic Stem Cell Transplantation in Late-Onset Krabbe Disease: No Evidence of Worsening Demyelination and Axonal Loss 4 Years Post-allograft. J Neuroimaging 28:252–255. https://doi.org/10.1111/jon.12502

32. Selleri S, Torchiana E, Pareyson D, et al (2000) Deletion of exons 11-17 and novel mutations of the galactocerebrosidase gene in adult- and early-onset patients with Krabbe disease. J Neurol 247:875–877. https://doi.org/10.1007/s004150070076

33. Wang C, Melberg A, Weis J, et al (2007) The earliest MR imaging and proton MR spectroscopy abnormalities in adult-onset Krabbe disease. Acta Neurol Scand 116:268–272. https://doi.org/10.1111/j.1600-0404.2007.00867.x

34. Hwang N, Kim S-M, Kim Y-G, et al (2024) Clinical feature, GALC variant spectrum, and genotype-phenotype correlation in Korean Krabbe disease patients: Multicenter experience over 13 years. Clin Genet 106:150–160. https://doi.org/10.1111/cge.14523

35. Luzi P, Rafi MA, Wenger DA (1996) Multiple mutations in the GALC gene in a patient with adult-onset Krabbe disease. Ann Neurol 40:116–119. https://doi.org/10.1002/ana.410400119

36. Malandrini A, D'Eramo C, Palmeri S, et al (2013) Peripheral neuropathy in late-onset Krabbe disease: report of three cases. Neurol Sci 34:79–83. https://doi.org/10.1007/s10072-012-0956-6

37. Fiumara A, Barone R, Arena A, et al (2011) Krabbe leukodystrophy in a selected population with high rate of late onset forms: longer survival linked to c.121G>A (p.Gly41Ser) mutation. Clinical Genetics 80:452–458. https://doi.org/10.1111/j.1399-0004.2010.01572.x

38. Debs R, Froissart R, Aubourg P, et al (2013) Krabbe disease in adults: phenotypic and genotypic update from a series of 11 cases and a review. J Inherit Metab Dis 36:859–868. https://doi.org/10.1007/s10545-012-9560-4

39. Romano A, De Simone R, Fasoli F, et al (2009) Selective white matter involvement in a patient with late onset Krabbe disease: MR, MR spectroscopy, and diffusion tensor study. J Neuroimaging 19:191–193. https://doi.org/10.1111/j.1552-6569.2008.00258.x

40. Harzer K, Knoblich R, Rolfs A, et al (2002) Residual galactosylsphingosine (psychosine) beta-galactosidase activities and associated GALC mutations in late and very late onset Krabbe disease. Clin Chim Acta 317:77–84. https://doi.org/10.1016/s0009-8981(01)00791-4

41. Kolodny EH, Raghavan S, Krivit W (1991) Late-onset Krabbe disease (globoid cell leukodystrophy): clinical and biochemical features of 15 cases. Dev Neurosci 13:232–239. https://doi.org/10.1159/000112166

42. Demaerel P, Wilms G, Verdru P, et al (1990) MR findings in globoid cell leucodystrophy. Neuroradiology 32:520–522. https://doi.org/10.1007/BF02426470

43. Tappino B, Biancheri R, Mort M, et al (2010) Identification and characterization of 15 novel GALC gene mutations causing Krabbe disease. Hum Mutat 31:E1894-1914. https://doi.org/10.1002/humu.21367

44. Lim SM, Choi B-O, Oh S-I, et al (2016) Patient fibroblasts-derived induced neurons demonstrate autonomous neuronal defects in adult-onset Krabbe disease. Oncotarget 7:74496–74509. https://doi.org/10.18632/oncotarget.12812

45. Africa L, Margollicci M, Salvatore S, et al (2017) Compound heterozygosity in the GALC gene in a late onset Iranian patient with spastic paraparesis, peripheral neuropathy and leukoencephalopathy. Neurol Sci 38:1721–1722. https://doi.org/10.1007/s10072-017-2986-6

46. Kapoor R, McDonald WI, Crockard A, Moseley IF (1992) Clinical onset and MRI features of Krabbe's disease in adolescence. J Neurol Neurosurg Psychiatry 55:331–332. https://doi.org/10.1136/jnnp.55.4.331
