# Supplementary material for: Very late-onset Krabbe disease with concomitant dementia: case description and a critical review of the literature
Source: Neurol Sci. 2026 Feb 13;47(3):257. doi: 10.1007/s10072-026-08836-5 (PMC12904878; doi:10.1007/s10072-026-08836-5)
Supplement: Supplementary file 2 — Supplementary file2 (PDF 57.7 KB) [file 10072_2026_8836_MOESM2_ESM.pdf]

Supplementary table 2. Table shows frequency of the pathogenic GALC variants in the cohort of KD patients from the literature.

***GALC* variant (NM_000153.4)**	**n**	% frequency (out of 132)
c.1901T>C	23	17,4
c.857G>A	17	12,9
c.1161+6532_polyA+9kbdel	13	9,8
c.334A>G	7	5,3
c.246A>G	7	5,3
c.169G>A	6	4,5
c.592G>A	4	3,0
c.195G>C	4	3,0
c.1901delT	3	2,3
c.1586C>T	3	2,3
c.1898C>T	3	2,3
c.1899del	2	1,5
c.1912 G>A	2	1,5
c.331G>A	2	1,5
c.1591C>T	2	1,5
c.956A>G	2	1,5
c.908+5G>A	1	0,8
c.461C>G	1	0,8
c.908+1G>A	1	0,8
c.952C>G	1	0,8
c.953C>G	1	0,8
c.884A>C	1	0,8
c.851G>A	1	0,8
c.760T>C	1	0,8
c.749T>C	1	0,8
c.977C>T	1	0,8
c.683_694delinsCTC	1	0,8
c.599C>A	1	0,8
exon 17 deletion	1	0,8
c.560A>T	1	0,8
c.1065G>C	1	0,8
c.461C>A	1	0,8
c.1911+1_1911+5del	1	0,8
c.1075_1084del	1	0,8
c.1468T>A	1	0,8
c.1534G>A	1	0,8
c.1655T>C	1	0,8
c.1658G>A	1	0,8
c.1687A>T	1	0,8
c.200C>T	1	0,8
c.415_417del	1	0,8
c.2041G>A	1	0,8

c.283_284del	1	0,8
c.326C>T	1	0,8
c.1065G>T	1	0,8
c.349A>G	1	0,8
c.391T>C	1	0,8
exon 6 deletion (c.583_621del)	1	0,8
